# Letrozole treatment of pubertal female mice results in activational effects on reproduction, metabolism and the gut microbiome

**DOI:** 10.1371/journal.pone.0223274

**Published:** 2019-09-30

**Authors:** Pablo Arroyo, Bryan S. Ho, Lillian Sau, Scott T. Kelley, Varykina G. Thackray

**Affiliations:** 1 Department of Biology, San Diego State University, San Diego, CA, United States of America; 2 Department of Obstetrics, Gynecology and Reproductive Sciences, University of California, San Diego, La Jolla, CA, United States of America; Peking University Third Hospital, CHINA

## Abstract

Polycystic ovary syndrome (PCOS) is a common endocrine disorder in reproductive-aged women that is comprised of two out of the following three features: hyperandrogenism, oligo- or amenorrhea, or polycystic ovaries. In addition to infertility, many women with PCOS have metabolic dysregulation that increases the risk of developing type 2 diabetes, hypertension, and non-alcoholic fatty liver disease. Changes in the gut microbiome are associated with PCOS and gut microbes may be involved in the pathology of this disorder. Since PCOS often manifests in the early reproductive years, puberty is considered to be a critical time period for the development of PCOS. Exposure to sex steroid hormones during development results in permanent, organizational effects, while activational effects are transient and require the continued presence of the hormone. Androgens exert organizational effects during prenatal or early post-natal development, but it is unclear whether androgen excess results in organizational or activational effects during puberty. We recently developed a letrozole-induced PCOS mouse model that recapitulates both reproductive and metabolic phenotypes of PCOS. In this study, we investigated whether letrozole treatment of pubertal female mice exerts organizational or activational effects on host physiology and the gut microbiome. Two months after letrozole removal, we observed recovery of reproductive and metabolic parameters, as well as diversity and composition of the gut microbiome, indicating that letrozole treatment of female mice during puberty resulted in predominantly activational effects. These results suggest that if exposure to excess androgens during puberty leads to the development of PCOS, reduction of androgen levels during this time may improve reproductive and metabolic phenotypes in women with PCOS. These results also imply that continuous letrozole exposure is required to model PCOS in pubertal female mice since letrozole exerts activational rather than organizational effects during puberty.

## Introduction

Polycystic ovary syndrome (PCOS) is a common endocrine disorder that affects approximately 10–15% of reproductive-aged women worldwide [1]. Diagnosis of PCOS is based on the 2003 Rotterdam Criteria that comprises at least two of the following features: hyperandrogenism, oligo- or amenorrhea and polycystic ovaries [2]. Women with PCOS have a higher incidence of infertility and pregnancy complications, as well as an increased risk for type 2 diabetes, hypertension and non-alcoholic fatty liver disease [3–5]. Although the etiology of PCOS is poorly understood, heritability and twin studies indicate that there is a strong genetic component that is likely polygenic [6–8]. Environmental factors, including prenatal exposure to androgens, may also play a role [9]. PCOS often manifests in the early reproductive years, suggesting that puberty may be a critical time for the onset of PCOS [9, 10].

Numerous animal models have been used to study PCOS [11–16] including rodent models based on the fact that many characteristic features of PCOS correlate with excess androgen levels [17]. Hyperandrogenism in these models is induced using treatment with testosterone, dihydrotestosterone (DHT; a non-aromatizable androgen) or letrozole (an aromatase inhibitor). Sex steroids were originally shown to result in both organizational and activational effects on behavior [18]. Organizational effects are considered to be permanent and often occur early in development although they can also occur later on in life. Activation effects, on the other hand, are transient and depend upon the continued presence of steroid hormone. While prenatal and early post-natal exposure of female rodents to androgens has been shown to result in organizational effects on the reproductive axis that are evident in adulthood [19, 20], it is not clear whether hyperandrogenism initiated during puberty results in organizational or activational effects.

Since DHT models do not recapitulate both reproductive and metabolic phenotypes of PCOS [21–25], we developed a pubertal mouse model of PCOS that uses letrozole, a nonsteroidal aromatase inhibitor, to limit the conversion of testosterone to estrogen, leading to increased testosterone and decreased estrogen levels. Letrozole treatment resulted in many reproductive hallmarks of PCOS including hyperandrogenism, acyclicity, polycystic ovaries, and elevated luteinizing hormone (LH) levels [26]. This model also exhibited metabolic dysregulation including weight gain, abdominal adiposity, increased fasting blood glucose (FBG) and insulin levels, and insulin resistance [27]. In addition, we demonstrated that changes in the gut microbiome were associated with letrozole treatment [28]. Furthermore, our studies showed that letrozole treatment did not alter food intake or energy expenditure, even though locomotion was decreased [27], suggesting that other mechanisms contribute to the metabolic dysregulation in this model.

Since puberty may be an important time period in the development of PCOS and it is currently unknown whether androgen excess induces activational effects during puberty, it is important to determine whether hyperandrogenic rodent models such as the letrozole model result in organizational or activational effects during puberty. To address this question, we investigated whether reproductive, metabolic and gut microbiome phenotypes induced by letrozole treatment of pubertal female mice persisted after letrozole removal or if they were transient. Removal of the letrozole pellet resulted in substantial recovery of reproductive and metabolic parameters as well as the composition of the gut microbiome, indicating that letrozole had predominantly activational effects during puberty. These results suggest that letrozole treatment needs to be maintained to model the effects of hyperandrogenism during puberty and adulthood. In addition, this study suggests that if exposure to excess androgens during puberty causes PCOS, reproductive and metabolic dysregulation may be improved if girls with PCOS receive treatment to decrease their androgen levels.

## Materials and methods

### PCOS mouse model

Twenty-three day old C57BL/6N female mice were purchased from Envigo. Mice were housed in a vivarium with a 12h:12h light/dark cycle (light period: 06.00–18.00). Mice were given ad libitum access to water and food (Teklad Global 18% Protein Extruded Diet, Envigo). All of the experiments were approved by the University of California San Diego Institutional Animal Care and Use Committee (Protocol S14011). At four weeks of age, 22 mice (with an average weight of 13.5 g) were subcutaneously implanted with either a placebo or continuous release letrozole pellet (3 mg, 50 μg/day, Innovative Research of America) (n = 10 placebo; n = 12 letrozole). Letrozole was purchased from Fitzgerald. After 5 weeks of treatment, the placebo and letrozole pellets were surgically removed. Mice were then monitored for an additional 8 weeks which resulted in a total of 13 weeks in the study design. Mice were weighted weekly throughout the experiment.

### Estrous cycle assessment

The estrous cycle of the mice was assessed during weeks 4–5 and 10–11 of the study. Estrous cycle stage was determined by light microscopy analysis of vaginal epithelial cells for seven days as previously described [29]. Proestrus consisted predominantly of nucleated epithelial cells; estrus of cornified epithelial cells; metestrus of cornified and nucleated epithelial cells and polymorphonuclear leukocytes; diestrus of predominantly polymorphonuclear leukocytes.

### Insulin tolerance test (ITT)

Mice were fasted for 5 hours with access to water. Tail vein blood was collected to measure fasting insulin levels. Blood glucose was measured using a handheld glucometer (One Touch Ultra2, LifeScan, Inc.). Fasting glucose levels were measured prior to time point 0. At time point 0, a single intraperitoneal injection of insulin (0.75 U/kg in sterile saline; Humulin R U-100) was administered. Glucose was measured subsequently at 15, 30, 45, 60, 90, and 120 minutes post administration of insulin.

### Fecal sample collection

Fecal samples were collected from mice prior to pellet implantation and each week afterwards until they were sacrificed. Fecal samples were stored at -80°C.

### Tissue collection and histology

At the end of the experiment, mice were euthanized with 2.5% isoflurane delivered with a precision vaporizer followed by a physical method. Terminal blood was collected through the inferior vena cava. Ovaries and parametrial fat pads were dissected and weighed. Ovaries were fixed overnight in 4% paraformaldehyde at 4°C and stored in ethanol. Fixed ovaries were serially sectioned at 10 μm and then stained with hematoxylin and eosin. The total number of non-overlapping corpora lutea and cystic follicles were counted from four sections from the middle of each ovary.

### Hormone assays

Hormone levels were assessed from weeks 4–5 and 10–13 of the study. Total testosterone was measured with 35 μL serum using a mouse ELISA (reportable range 10–1600 ng/dL) and LH was measured with 60 μL serum using a radioimmunoassay (reportable range 0.016–4.0 ng/mL) by the University of Virginia Center for Research in Reproduction Ligand Assay and Analysis Core. Insulin was measured with 10 μL serum using a mouse ELISA (ALPO) by the University of California, Davis Mouse Metabolic Phenotyping Center.

### Next generation sequencing and bioinformatics analysis of 16S rRNA genes

DNA was extracted from fecal samples using the DNeasy PowerSoil Kit (Qiagen). 16s ribosomal RNA genes were amplified by PCR using 16S primers (515F and 806R) that target the V4 hypervariable region [30]. The reverse primers also contained unique 12 base pair Golay barcodes that were incorporated into the PCR amplicons [31]. The resulting amplicons were submitted to The Scripps Research Institute NGS core facility where they were used to prepare libraries that were sequenced on an Illumina MiSeq as previously described [28]. Sequences were analyzed using the open source software pipeline Quantitative Insights Into Microbial Ecology (QIIME version 1.9.1) [32]. The 16S rRNA sequence data was demultiplexed and then quality filtered using default QIIME parameters with the split_libraries.py script. This resulted in an average of 20,890 sequences per sample. The 16S rRNA sequences generated in this study were deposited into the European Nucleotide Archive (Study Accession Number PRJEB31499). Sequences were clustered using a denovo operational taxonomic unit (OTU) picking approach (pick_de_novo_otus.py) with usearch. Sequences were assigned to OTUs with an assumed 97% threshold of pairwise identity for bacterial species by comparison with the Greengenes reference database using the RDP classifier. OTUs present in less than 25% of the samples were discarded from the dataset to minimize the effect of spurious, low abundance sequences using the filter_otus_from_otu_table.py script resulting in 1451 OTUs. Faith’s Phylogenetic Diversity (Faith’s PD) [33], which measures the biodiversity of an ecosystem by calculating the total branch lengths on a phylogenetic tree of all members of the ecosystem, was calculated using the alpha_diversity.py script. The R package phyloseq (1.26.0) was used to compute weighted UniFrac distances [34, 35]. DESeq2 [36] (version 1.14.1) in the microbiomeSeq package was used to identify bacterial genera that were differentially abundant between placebo and letrozole-treated mice.

### Statistical analysis

Data is expressed as the mean ± standard error of the mean (SEM) for each group. Data residuals were checked for normality and data underwent Box Cox transformation if residuals were not normal. If transformation did not result in normality, a non-parametric test was used. Group differences were analyzed by Student t-test, Welch t-test or Wilcoxon rank-sum test. Insulin tolerance was analyzed with a repeated measures (RM) ANOVA. Faith’s PD was analyzed with a simple linear regression model (LM) and a RM-ANOVA. Weighted UniFrac was analyzed through an Analysis of Similarities test (ANOSIM). Statistical calculations were performed with the R statistical package (version 3.5.1). Statistical significance was defined as p < 0.05. Data is available at https://doi.org/10.6084/m9.figshare.9502403.v1.

## Results

### Letrozole removal resulted in testosterone and LH levels similar to placebo mice

**Fig 1:**
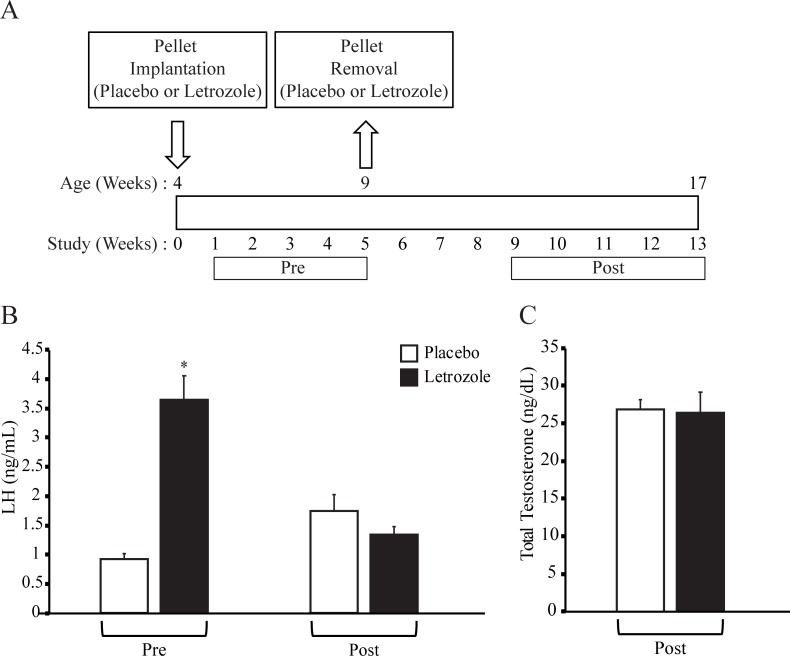
Letrozole removal resulted in testosterone and LH levels similar to placebo female mice. Schematic of study design: female mice were implanted with placebo or letrozole pellets at 4 weeks of age (week 0) and the pellets were removed at 9 weeks of age (week 5) (A). Reproductive and metabolic phenotypes were evaluated before (Pre) and after (Post) pellet removal. Luteinizing hormone (LH) was measured in placebo and letrozole-treated female mice (n = 10 placebo, n = 12 letrozole) (B). LH levels were measured in week 5 before pellet removal (Pre) and in week 11 (Post). Serum total testosterone was measured in placebo and letrozole-treated mice in week 13 (Post; n = 4 placebo, n = 12 letrozole) (C). Welch t-test was used because the variances between groups was not equal; * p < 0.05.

As illustrated in Fig 1A, female mice were implanted with placebo or letrozole pellets at 4 weeks of age (week 0), the pellets were removed at 9 weeks of age (week 5) and the study concluded 2 months later (week 13). Removal of the placebo pellet controlled for the effect of surgical removal of the pellet. Since testosterone levels could not be measured prior to pellet removal due to the amount of serum needed for the assay, we measured LH levels as a proxy for testosterone. Similar to previously published studies [26], 5 weeks of letrozole treatment resulted in elevated LH levels compared to placebo treatment (Fig 1B). Serum LH and total testosterone levels were measured at the end of the experiment (8 weeks after removal of letrozole or placebo pellets). There was no difference in LH and testosterone levels of placebo- and letrozole-treated mice after pellet removal (Fig 1C).

### Letrozole removal resulted in resumption of estrous cycling and ovulation

**Fig 2:**
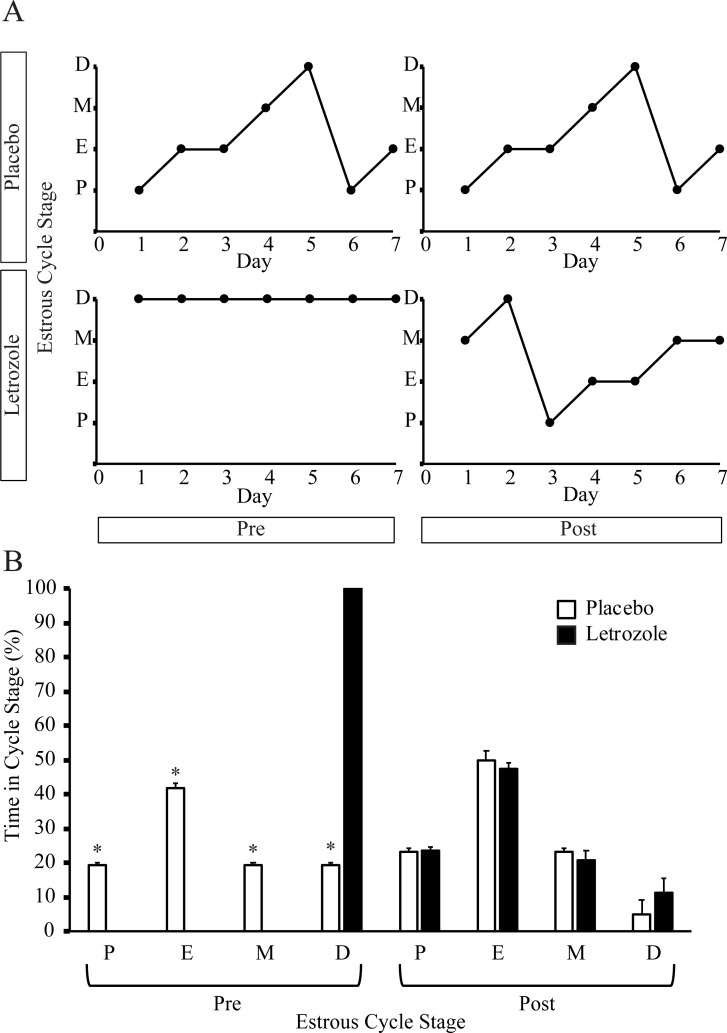
Letrozole removal resulted in resumption of estrous cycling. Estrous cycling was evaluated in placebo and letrozole-treated female mice via vaginal cytology. Vaginal smears were collected during weeks 4–5 (Pre; n = 6 placebo, n = 10 letrozole) and during weeks 10–11 (Post; n = 6 placebo, n = 8 letrozole). Representative estrous cycles are shown for placebo and letrozole mice before (Pre) and after (Post) pellet removal (A). The stages of the estrous cycle were represented as Proestrus (P), Estrus (E), Metestrus (M) and Diestrus (D). The time spent in each stage of the estrous cycle was graphed for placebo and letrozole mice before (Pre) and after (Post) pellet removal (B). Wilcoxon rank sum test, a non-parametric test was used; * p < 0.05.

**Fig 3:**
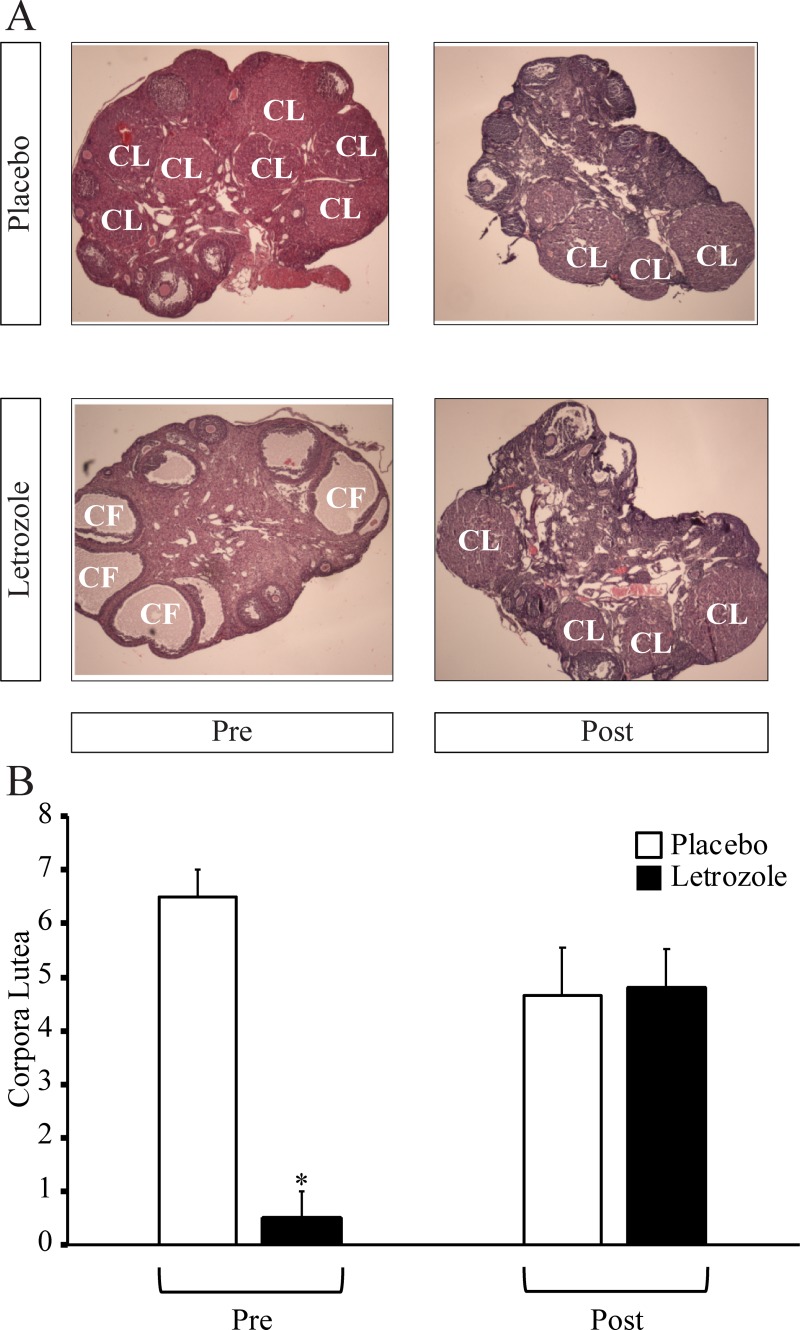
Letrozole removal resulted in ovaries with corpora lutea that lacked cystic follicles. Representative ovaries with corpora lutea (CL) and cystic follicles (CF) are shown at 40X magnification for placebo and letrozole-treated mice before (Pre) and after (Post) pellet removal (A). The number of CL in the ovaries of letrozole and placebo mice before (Pre; n = 3 placebo, n = 3 letrozole) and after (Post; n = 3 placebo, n = 6 letrozole) pellet removal were quantified (B). Wilcoxon rank sum test, a non-parametric test was used; * p < 0.05. Ovaries from placebo and letrozole-treated mice before pellet removal were obtained from a previously published cohort [28].

Similar to previous studies [26, 28], letrozole treatment of female mice resulted in acyclicity with an arrest in diestrus (Fig 2A). Further analysis of the estrous cycle revealed that letrozole-treated mice resumed cycling after removal of the letrozole pellet and that placebo- and letrozole-treated mice spent a similar amount of time in all four estrous cycle stages after pellet removal (Fig 2A and 2B). As previously reported [26, 28], 5 weeks of letrozole treatment resulted in ovaries that lacked corpora lutea (Fig 3B) and contained, on average, 6±2 cystic follicles. After pellet removal, the ovaries of letrozole-treated mice had a similar number of corpora lutea compared to placebo mice and did not contain any cystic follicles (Fig 3B). These results indicate that restoration of estrous cycling and ovulation occurred 7–8 weeks after removal of the letrozole pellet.

### Letrozole removal resulted in weight gain and abdominal adiposity similar to placebo mice

**Fig 4:**
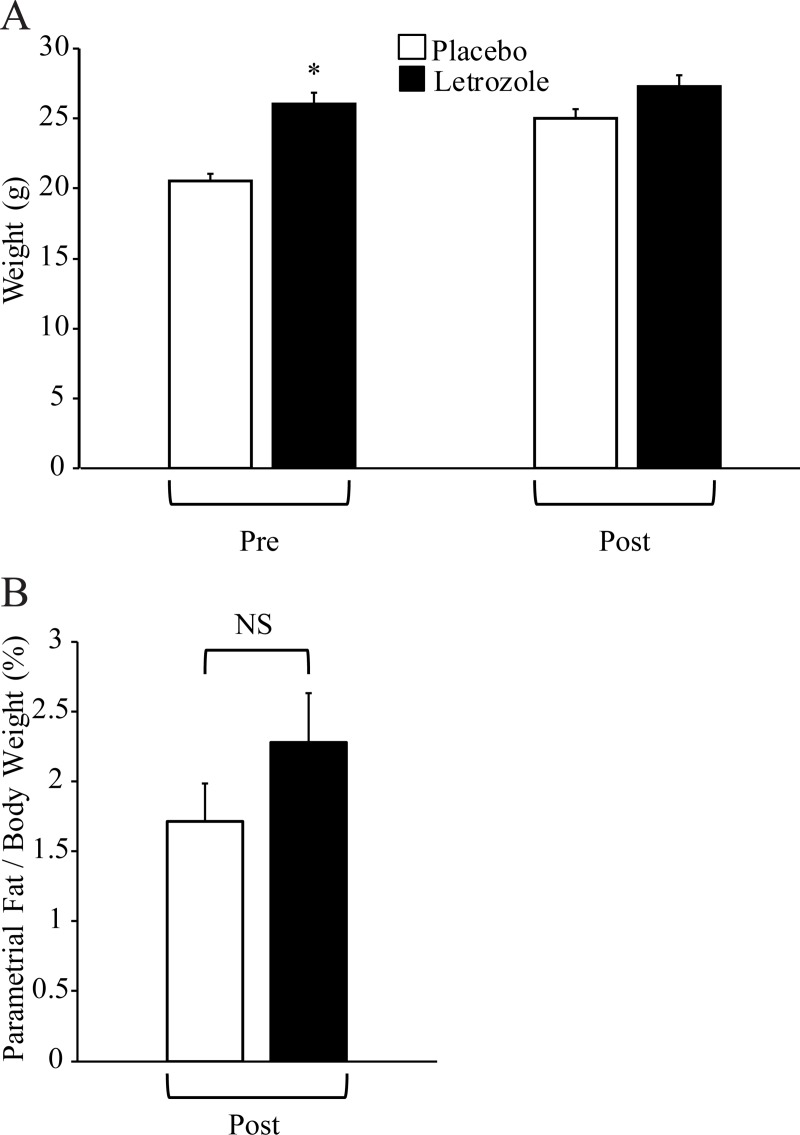
Letrozole removal resulted in weight and abdominal adiposity similar to placebo mice. Body weight was measured for placebo and letrozole-treated mice before (Pre; week 5) and after (Post; week 13) pellet removal (n = 10 placebo, n = 12 letrozole) (A). Abdominal adiposity was evaluated by measuring parametrial fat relative to total body weight for placebo and letrozole-treated mice after (Post; week 13) (B). Student t-test was used, * p < 0.05.

In previous findings, letrozole treatment of female mice resulted in weight gain and abdominal adiposity compared to placebo mice [26, 28]. In this cohort, 5 weeks of letrozole treatment also resulted in increased body weight (Fig 4A). When body weight and parametrial fat were measured at the end of the experiment, placebo and letrozole-treated mice had similar body weights and amounts of parametrial fat relative to body weight post-pellet removal (Fig 4A and 4B).

### Letrozole removal resulted in a minimal metabolic phenotype

**Fig 5:**
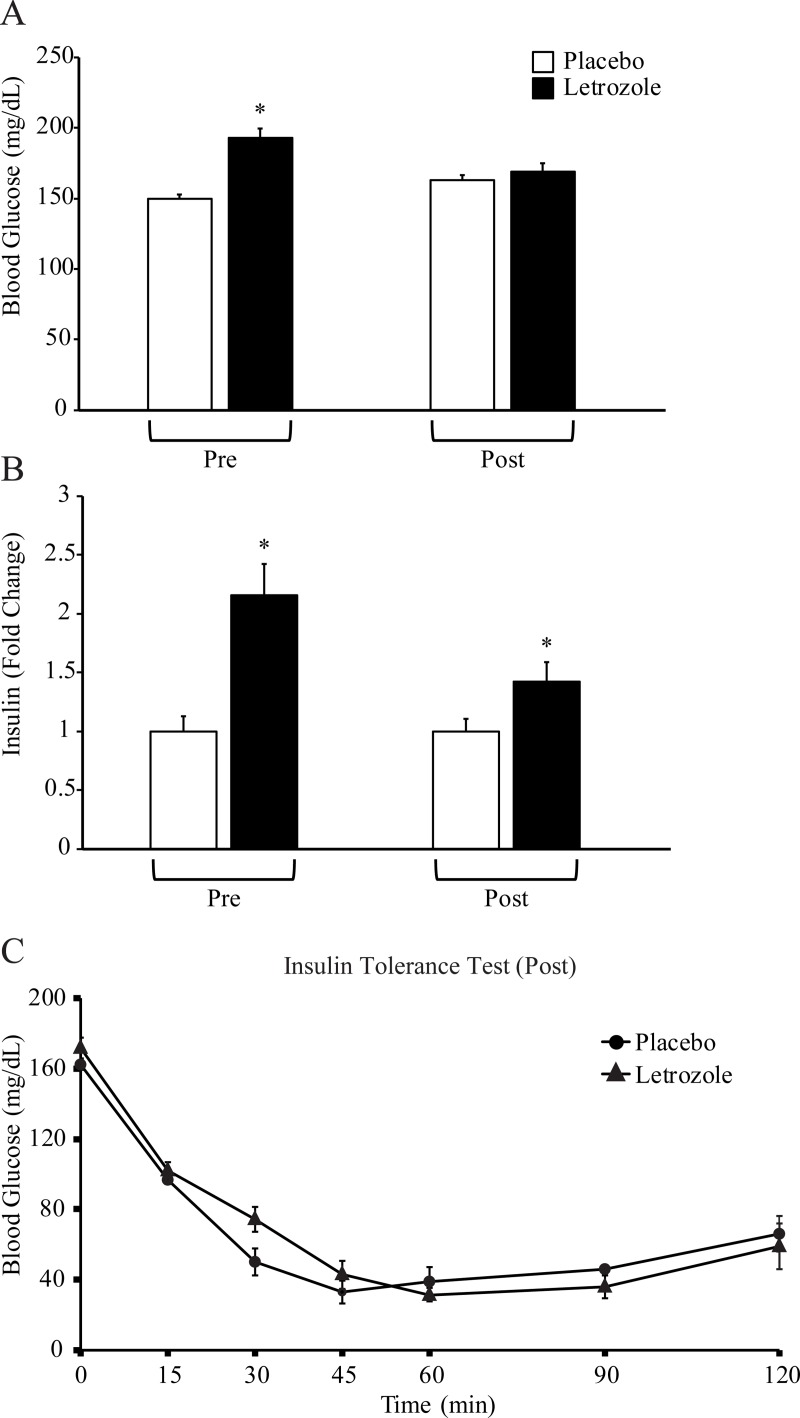
Letrozole removal resulted in fasting blood glucose levels and insulin tolerance similar to placebo mice. Fasting blood glucose (A) and insulin levels (B) were measured for placebo and letrozole-treated mice before (Pre; week 5) and after (Post; week 12) pellet removal (n = 8 placebo, n = 8 letrozole). Removal of the letrozole pellet resulted in normalized fasting blood glucose levels and less elevation of insulin levels compared to before pellet removal. Student t-test was used, * p < 0.05. An insulin tolerance test (ITT) was performed on placebo and letrozole-treated mice after pellet removal (Post; week 12) (C) Removal of the letrozole pellet resulted in a lack of insulin resistance. A repeated measures ANOVA was used for comparing differences between placebo and letrozole mice over time, p < 0.05.

Previous studies showed that letrozole treatment of female mice resulted in increased FBG and insulin levels and insulin resistance [27]. Prior to pellet removal, FBG and insulin levels also increased in this cohort of letrozole-treated mice compared to placebo mice (Fig 5). Eight weeks after pellet removal, FBG levels were similar in placebo- and letrozole-treated mice (Fig 5A). In addition, fasting blood insulin levels were less elevated in letrozole-treated mice after pellet removal than before pellet removal (1.4 fold vs. 2.2 fold) (Fig 5B). Importantly, insulin sensitivity was restored in letrozole-treated mice compared to placebo mice 8 weeks after removal of the letrozole pellet (Fig 5C).

### Letrozole removal resulted in alpha diversity of the gut microbiome similar to placebo mice

**Fig 6:**
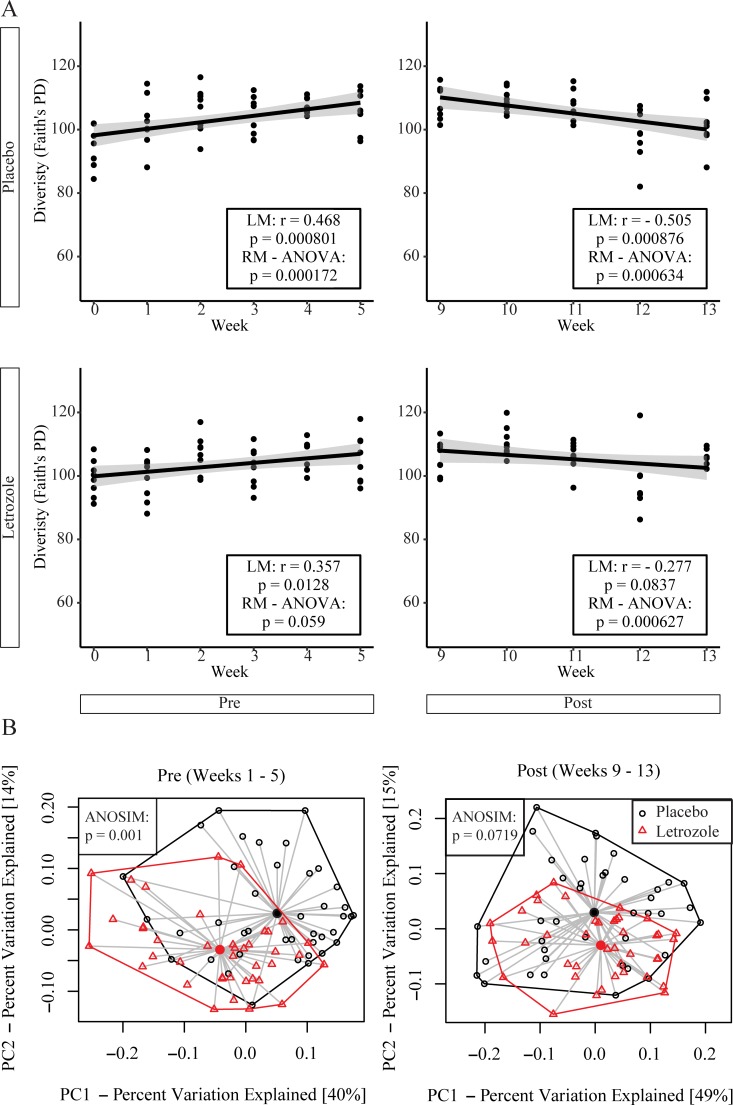
Letrozole removal resulted in gut microbial diversity similar to placebo mice. Alpha diversity of the gut microbiome according to Faith’s phylogenetic diversity (Faith’s PD) estimate was graphed over time for placebo and letrozole-treated female mice before (Pre; weeks 0–5) and after (Post; weeks 9–13) pellet removal (n = 10 placebo, n = 12 letrozole) (A). Results of simple linear regression model (LM) and Repeated Measures (RM) ANOVA are in the box inset, while the gray shaded area indicates the 95% confidence interval for the line of best fit. Beta diversity was estimated using weighted UniFrac distances and a Principal Coordinates Analysis (PCoA) was used to demonstrate changes in the gut bacterial community in placebo and letrozole-treated mice before (Pre; weeks 1–5) and after (Post; weeks 9–13) pellet removal (B). Centroid of placebo samples (black solid circle) and centroid of letrozole samples (red solid circle) are indicated on the graph. The proportion of variance explained by each principal coordinate axis (PC) is shown with the corresponding axis. Analysis of Similarity (ANOSIM) test is shown in the box inset.

Since changes in the gut microbiome were reported to correlate with PCOS in both women and in rodent models [28, 37–39], we investigated whether letrozole treatment resulted in organizational or activational effects on the composition of the gut microbiome. The species richness (alpha diversity) of the gut microbiome in placebo and letrozole-treated female mice was analyzed using Faith’s PD estimate before (weeks 1–5) and after (weeks 9–13) pellet removal (Fig 6A). Similar to a previous report [28], placebo mice showed a significant positive correlation with alpha diversity during the first 5 weeks of the study that corresponded with puberty. In contrast, letrozole treatment of pubertal female mice did not result in a significant change in alpha diversity over time (RM-ANOVA: p = 0.059) (Fig 6A). After pellet removal, placebo mice did not demonstrate a positive correlation with alpha diversity over time and letrozole mice demonstrated similar alpha diversity to placebo-treated mice (Fig 6A).

### Letrozole removal resulted in beta diversity of the gut microbiome similar to placebo mice

In addition to examining alpha diversity, UniFrac analyses were used to compare the similarity of gut microbial communities (beta diversity) of placebo and letrozole-treated mice (Fig 6B). Weighted UniFrac takes into account the abundance of bacterial OTUs in each fecal sample. The weighted UniFrac results were visualized using Principal Coordinates Analysis (PCoA) that displays a multi-dimensional matrix in low dimensional space (2D plot). Similar to our previous study [28], letrozole treatment (weeks 1–5) resulted in significant differences in the overall bacterial community composition compared to placebo mice (ANOSIM: p = 0.001). After pellet removal, UniFrac analysis showed that there was no significant different in the gut bacterial community composition between placebo and letrozole mice (ANOSIM: p = 0.0719).

### Letrozole removal resulted in fewer differential gut bacterial abundances between placebo and letrozole mice

**Fig 7:**
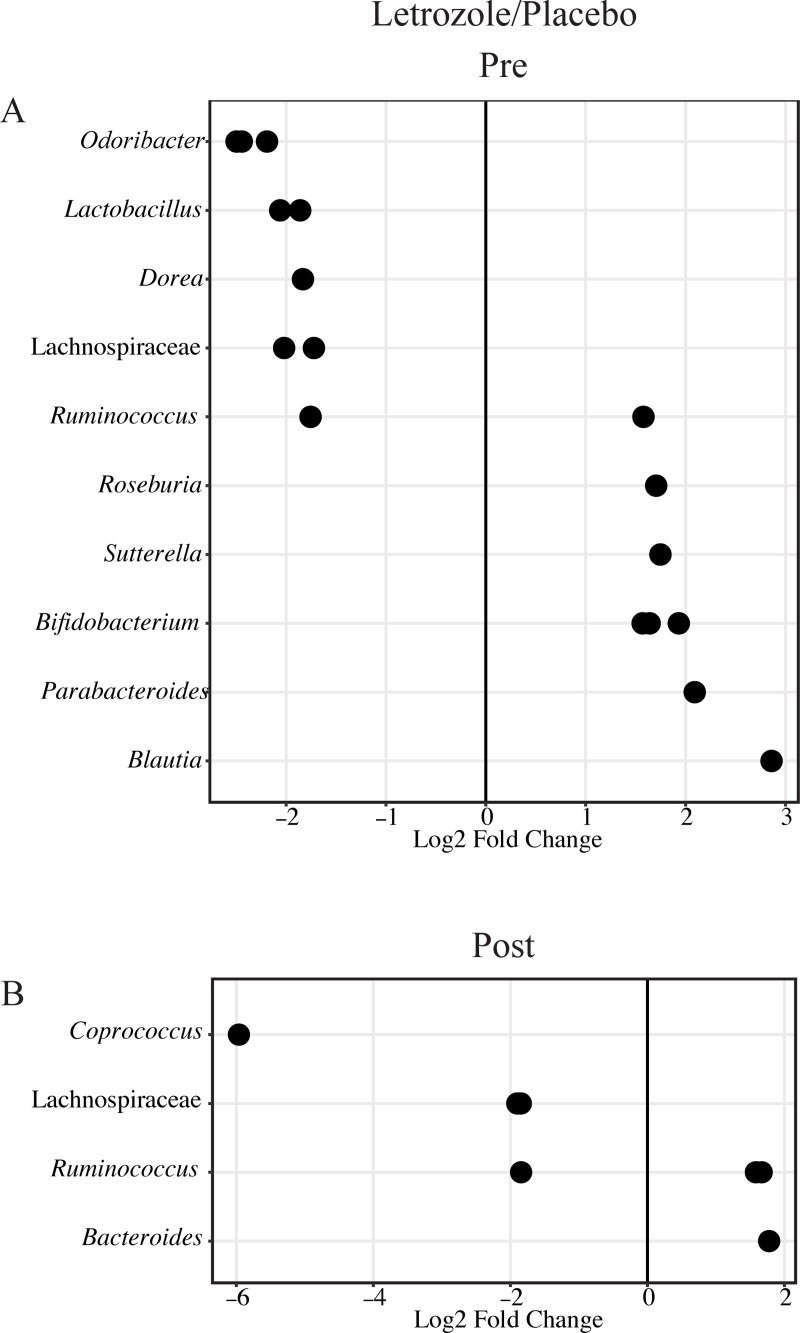
Letrozole removal resulted in fewer differentially abundant bacterial genera in placebo versus letrozole mice. DESeq2 differential abundance results were expressed as log2 fold change to compare placebo- and letrozole-treated female mice before (A) and after pellet removal (B). Positive log2 fold change represents bacterial genera increased in letrozole relative to placebo mice. Negative log2 fold change represents bacterial genera increased in placebo relative to letrozole mice. p < 0.05.

In addition to analyzing the effect on overall bacterial diversity, we used DESeq2 to assess the number of bacterial taxa that were differentially abundant in mice treated with placebo versus letrozole. Ten bacterial genera or family (if genera unknown) were identified as having differential relative abundances in letrozole-treated mice compared to placebo-treated mice during weeks 1–5 of treatment (Fig 7A). Similar to previous studies [28, 40, 41], the relative abundance of *Lactobacillus*, *Dorea*, Lachnospiraceae spp., *Ruminococcus*, *Roseburia*, *Sutterella*, *Bifidobacterium*, *Parabacteroides*, and *Blautia* were altered in letrozole-treated mice compared to placebo mice. Interestingly, the number of bacterial genera that had a differential relative abundance in letrozole compared to placebo-treated mice decreased from 10 to 4 genera two months after pellet removal (Fig 7B).

## Discussion

Our study clearly showed that letrozole treatment of pubertal female mice resulted in activational effects on the reproductive axis. As demonstrated in previous studies [26, 28], 5 weeks of letrozole treatment resulted in hallmarks of PCOS including elevated testosterone and LH levels, acyclicity, anovulation (indicated by a lack of corpora lutea in the ovaries), and the appearance of cystic ovarian follicles. Discontinuation of letrozole treatment resulted in recovery of testosterone and LH levels after 2 months as well as a resumption of estrous cycling and ovulation (indicated by the appearance of corpora lutea and a lack of cystic follicles). These results are in agreement with a previous study showing that letrozole-treated female mice were infertile compared to placebo mice but that the reproductive deficit was reversible [26]. In the previous study, placebo- and letrozole-treated females were paired with a male mouse 4 months after the expiration of the letrozole pellet. All control and letrozole-treated mice gave birth to a litter, with no difference in time to birth (latency) or litter size [26]. Given the transient effects of letrozole on reproduction, these results support the idea that hyperandrogenism does not result in permanent, organizational effects on the female reproductive axis during puberty. The pubertal letrozole model stands in contrast to organizational effects of androgens during the prenatal period which resulted in permanent changes in the brain including alterations in GnRH neurons and anxiety-like behavior [19, 20, 42].

In addition to studying the activational versus organizational effects of letrozole on reproduction, we also investigated letrozole effects on metabolism. As previously demonstrated [27, 28], letrozole treatment of pubertal female mice resulted in many features of metabolic dysregulation observed in women with PCOS including weight gain, increased FBG and insulin levels, and insulin resistance. Interestingly, letrozole removal resulted in marked improvement in all of the metabolic parameters examined. Two months after removal of the letrozole pellet, placebo and letrozole-treated mice had statistically similar body weights, abdominal adiposity, FBG levels, and insulin tolerance. In addition, fasting insulin levels were reduced in letrozole-treated mice compared to placebo mice after removal of the letrozole pellet versus prior to pellet removal (1.4 vs. 2.4 fold). These results indicated that letrozole treatment of pubertal female mice resulted in predominantly activational effects on metabolism. However, the slight elevation in insulin levels 2 months after removal of the letrozole pellet suggested that metabolism did not completely recover during this time frame. Given studies demonstrating that long-term exposure to androgens may result in permanent effects on metabolism [43], it would be interesting to further investigate how quickly metabolism normalizes after discontinuation of letrozole or androgen treatment and whether long-term androgen exposure can result in organizational effects on metabolism during adulthood.

Since studies have shown that obesity-induced alterations to the gut microbiome persist even when metabolic parameters revert back to normal upon dieting [44], we investigated whether letrozole treatment of pubertal female mice resulted in organizational or activational effects on the gut microbial community. Similar to our previous study [28], our results showed that letrozole treatment was associated with changes in the gut microbiome including a decrease in bacterial species richness (alpha diversity) and a change in the overall bacterial community composition (beta diversity). Moreover, we demonstrated that the relative abundance of specific bacterial genera including *Odoribacter*, *Lactobacillus*, *Dorea*, Lachnospiraceae spp., *Ruminococcus*, *Roseburia*, *Sutterella*, *Bifidobacterium*, *Parabacteroides*, and *Blautia* were altered with letrozole treatment. These changes in the gut microbiome of the letrozole-induced PCOS mouse model were consistent with changes identified in the gut microbiome of women with PCOS compared to healthy controls [37–39, 45]. Similar to host metabolism, removal of the letrozole pellet resulted in substantial recovery of the gut microbiome with regards to biodiversity, community composition and relative abundance of specific bacterial genera, indicating that letrozole exerted predominantly activational effects on the gut microbiome. Since letrozole treatment during puberty exerted activational effects on the gut microbiome, and these changes appear to be tightly linked with changes in reproduction and metabolism, our results suggest that therapeutic approaches targeting the gut microbiome may be worth developing to treat PCOS. It should be noted that the 16S rRNA gene sequencing employed in this study provided information about the taxonomic composition of the gut microbiome but not about functional changes. Given that a longer duration of androgen exposure may result in permanent effects on metabolism [43], it would be informative to employ metagenomic, transcriptomic or metabolomics analyses to understand whether exposure to excess androgens for a short or long duration can result in organizational or activational effects on gut microbial function.

In summary, our study demonstrated that letrozole treatment of pubertal female mice resulted in mostly activational effects on reproduction, metabolism and gut microbiome. Although organizational effects of steroids during puberty have been reported in rodents [46], our results indicate that exposure to excess androgens during puberty as a result of treatment with the aromatase inhibitor, letrozole did not result in substantial organizational effects on the female reproductive axis, metabolism or gut bacterial diversity and composition. Given that puberty may be a critical time period in the development of PCOS, this study supports the idea that reducing hyperandrogenism during puberty may be an important therapeutic tool to improve reproductive and metabolic dysfunction in girls predisposed to develop PCOS. Moreover, although testosterone, DHT and letrozole are commonly employed to induce PCOS-like characteristics in pubertal or adult female rodents, it is not well understood whether androgen excess during these time periods results in organizational or activational effects. Indeed, several recent studies have assumed that letrozole treatment of pubertal female rodents causes organizational effects [47–50]. Given that our study demonstrated that letrozole treatment exerted mostly activational effects during puberty, this finding implies that letrozole treatment needs to be present for the entire duration of the study to effectively model PCOS. Additional studies should be performed with other hyperandrogenic rodent models to determine whether excess androgen exposure during puberty or adulthood result in organizational or activational effects on host physiology or the gut microbiome.
